# C1 Lateral Mass Screw Insertion Caudally From the C2 Nerve Root to Avoid Craniocervical Fusion in a Patient With Atlantoaxial Subluxation Associated With Ponticulus Posticus: A Case Report

**DOI:** 10.7759/cureus.73478

**Published:** 2024-11-11

**Authors:** Satoshi Hattori, Keiji Wada, Futoshi Watanabe, Satoru Matsutani

**Affiliations:** 1 Spinal Surgery, Hachioji Spine Clinic, Hachioji, JPN

**Keywords:** 3-dimensional computed tomography angiograpny, atlantoaxial fixation, atlantoaxial subluxation, c1 lateral mass screw, c1 pedicle screw, high-riding vertebral artery, image-guided navigation, occipitocervical fusion, poticulous posticus

## Abstract

This report describes the case of a 78-year-old female patient with a rare complex upper cervical spine disorder combined with atlantoaxial subluxation (AAS), ponticulus posticus (PP), and high-riding vertebral artery (HRVA), treated with posterior C1-C3 screw fixation. To avoid vertebral artery injury during screw insertion, a C1 lateral mass screw (LMS) on the PP side was inserted from the caudal side of the C2 nerve root. Preoperative three-dimensional CT angiography is important for selecting the optimal posterior screw entry point and trajectory among several screw options. C1 LMS insertion from the caudal side of the C2 nerve root may be an alternative screw trajectory in the PP with vertebral artery running variation.

## Introduction

Ponticulus posticus (PP) is a common anatomical variant of the atlas with a bony bridge between the posterolateral portion of the superior articular process and the lateral portion of the posterior arch of the atlas, encircling all or the part of the vertebral artery (VA). Its prevalence is reported to be 5-55.7% and varies widely depending on the region and the methods of the analysis used in the studies (6.2-19% in East Asia) [1-3]. PP is considered to have few clinical manifestations, although several studies have shown a significant association between PP and headache, migraine, dizziness, and vertigo (the so-called "PP syndrome") [4,5]. 

Recently, the safety and feasibility of inserting a C1 lateral mass screw (LMS) in patients with PP have been watched with interest by spine surgeons [6-8], as PP itself is a common variant and posterior atlantoaxial fixation using the C1 LMS via the posterior arch (C1 pedicle screw (PS)) has been used more frequently to avoid massive bleeding, less biomechanical stability, and C2 nerve root irritation with the Goel-Harms approach [9-12].

The VA of the PP patients is usually smaller than the normal VA, and its running course may be displaced due to the bony foramen [2]. In addition, the PP may be missed on the preoperative lateral radiograph or misinterpreted as a wide posterior atlantal arch, which may lead to an unexpected VA injury during screw insertion [8,13]. On the other hand, Lee et al. reported that the thickness of the posterior arch below the PP was the same as in non-PP patients, and the presence of PP may not necessarily be a contraindication to the C1 LMS insertion through the posterior arch [14]. Thus, there remains considerable controversy regarding the selection of an optimal surgical fixation construct for atlantoaxial subluxation (AAS) with PP, depending on the anatomical variations in each case.

We present a case of AAS associated with VA running variation due to PP and high-riding VA (HRVA), in which we performed C1-C3 fusion with C1 LMS inserted from the caudal side of the C2 nerve root on the PP side, as proposed by Wada et al. in 2016 [15], to avoid VA injury with C1 PS and massive bleeding and C2 nerve root irritation with conventional Goel-Harms C1 LMS insertion. Finally, we were able to avoid occipitocervical fusion (OCF) in an elderly patient. This case study highlights the importance of selecting the optimal trajectory for C1 LMS insertion in these specific craniocervical junction pathologies, depending on the anatomical relationship between the VA and surrounding bony structures, as assessed by preoperative three-dimensional (3D) computed tomography angiography (CTA). C1 LMS insertion from the caudal side of the C2 nerve root may become a useful trajectory option among several approaches to the atlas.

## Case presentation

History and examination

**Figure 1:**
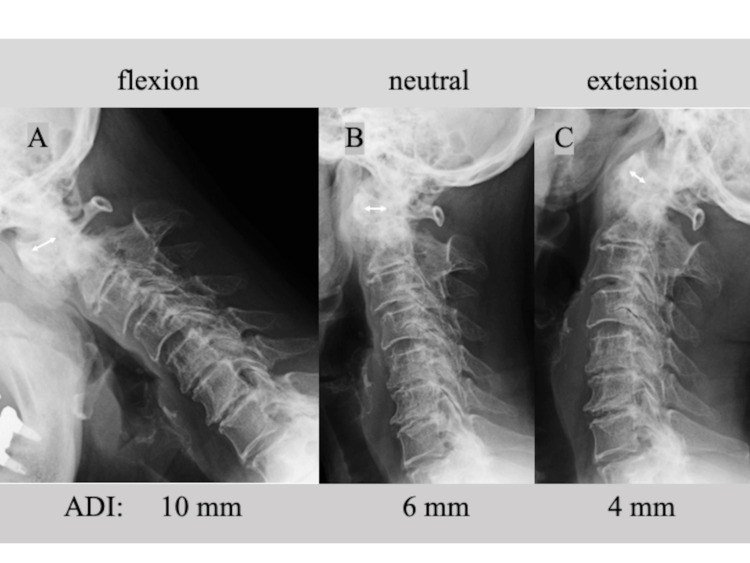
Lateral cervical spine plain X-rays at initial presentation showing an increase in the atolantodental interval of (A) 10 mm in flexion, (B) 6 mm in neutral, and (C) 4 mm in extension (white double-headed arrows). ADI: atlantodental interval

**Figure 2:**
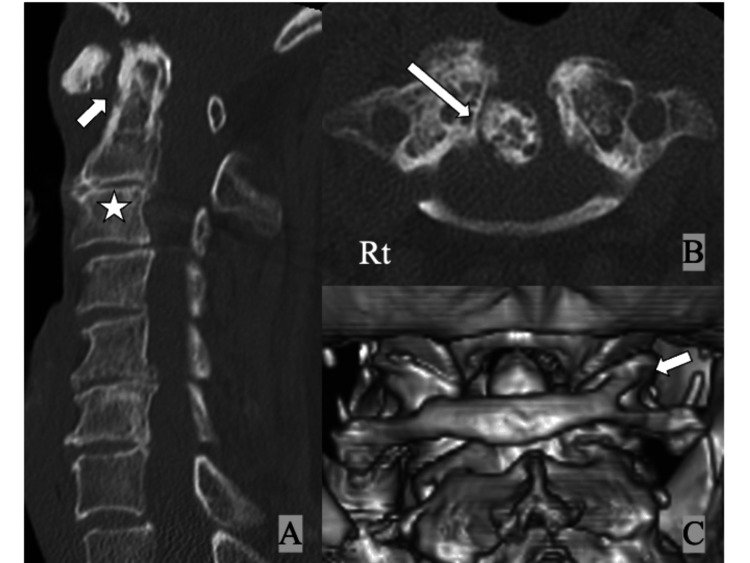
CT of the cervical spine at initial presentation showing (A) increased atlantodental interval (white arrow) and spontaneous fusion of the axis and C3 (white star), (B) osteoarthritic change with bony spur around the dens (white long arrow), and (C) the bony bridge between the posterior arch and the superior facet of the atlas on the right side (complete "ponticulus posticus", white arrow).

**Figure 3:**
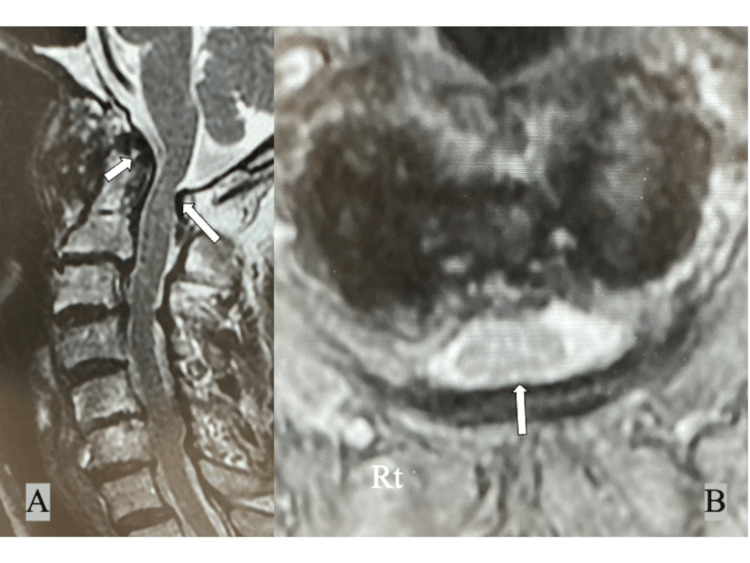
Preoperative MRI of the cervical spine showing (A) synovitis around the dens (white arrow) and (A, B) mild spinal cord compression between the dens and the posterior atlantal arch (white long arrows).

A 78-year-old female patient presented to the outpatient department with persistent posterior neck pain, occipitalgia, and numbness in both hands for several years. Neurological examination was almost normal except for slightly increased bilateral patellar tendon reflexes (manual muscle test: 5/5, sensory disturbance (-), gait disturbance (-), dexterity disturbance (-), bladder bowel disturbance (-)). Lateral X-rays of the cervical spine showed an increased atlantodental interval (ADI) of 6 mm in neutral, 10 mm in flexion, and 4 mm in extension (Figure 1). Computed tomography (CT) showed bony spurs around the dens, increased ADI, and spontaneous fusion of C2 and C3 (Figures 2A, 2B); 3D CT showed the bony bridge between the posterior arch and the superior facet of the atlas on the right side (Figure 2C). She was diagnosed with cervical osteoarthritis (OA) and AAS. 

**Figure 4:**
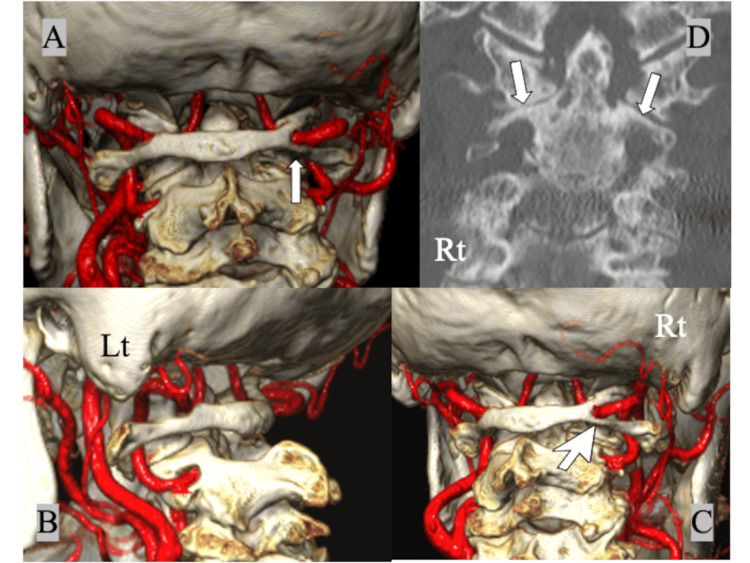
Preoperative 3D CTA showing (A) the bony bridge completely encircling the right VA on the right side (complete ponticulus posticus, white arrow), (B) a normal running course of the left VA on the VA groove, (C) a narrower posterior atlantal arch below the ponticulus posticus (white large-head arrow), and (D) bilateral high riding VAs with the thin C2 isthmus (white arrows). 3D: three dimensional; CTA: computed tomography angiography; VA: vertebral artery

Preoperative magnetic resonance imaging (MRI) showed synovitis around the dens and mild spinal cord compression between the dens and the posterior atlantal arch (Figure 3), and 3D CTA showed the bony bridge completely encircling the right VA ("right complete PP", Figures 4A, 4C). The right VA was slightly displaced caudally and posteriorly compared to the normal course of the left VA, and the thickness of the posterior arch below the PP was significantly less than the left (Figures 4B, 4C). The posterior height of the C1 lateral mass was slightly narrower on the PP side than on the left side due to the OA change. In addition, the height of the C2 isthmus was less than 5 mm bilaterally and HRVA was present bilaterally (Figure 4D). Definitive diagnosis of PP on plain lateral radiographs was difficult due to advanced OA changes around the atlantoaxial joint.

Treatment and follow-up

To achieve rigid posterior screw fixation without OCF, we performed a C1-C3 posterior fusion with C1 LMS inserted from the caudal side of the C2 nerve root on the right (PP side) and via the posterior arch on the left (C1 PS), the right C2 laminar screw (LS), and bilateral C3 PS. Reduction of the subluxated atlantoaxial joint was achieved by sublaminar taping and Brooks iliac bone grafting. All screws were inserted safely under navigational guidance linked to the O-Arm II system (Medtronic plc, Dublin, Ireland). The operative time was 154 minutes and intraoperative blood loss was 180 ml. 

**Figure 5:**
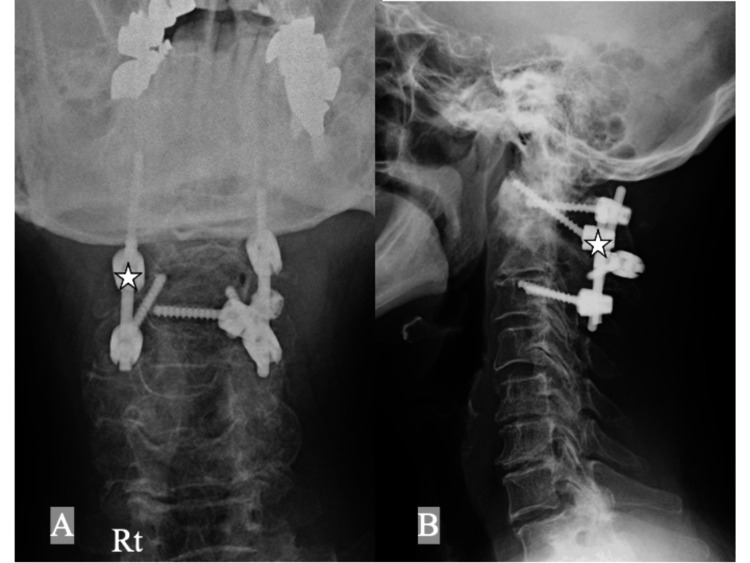
Postoperative cervical spine plain X-rays, (A) anteroposterior and (B) lateral views, of the cervical spine after surgery showing a C1-C3 posterior fusion with C1 lateral mass screw inserted from the caudal side of the C2 nerve root on the right (ponticulus posticus side, white stars) and via the posterior arch on the left (C1 pedicle screw), the right C2 laminar screw, and bilateral C3 pedicle screws.

**Figure 6:**
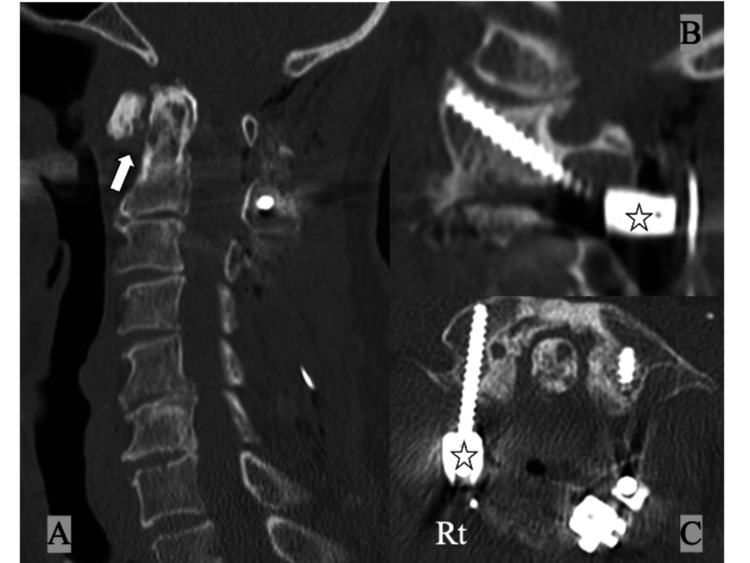
Postoperative CT of the cervical spine showing (A) good reduction of the atolantoaxial subluxation (white arrow), (B, C) the C1 lateral mass screw inserted from the caudal side of the right C2 nerve root (ponticulus posticus side, white stars).

**Figure 7:**
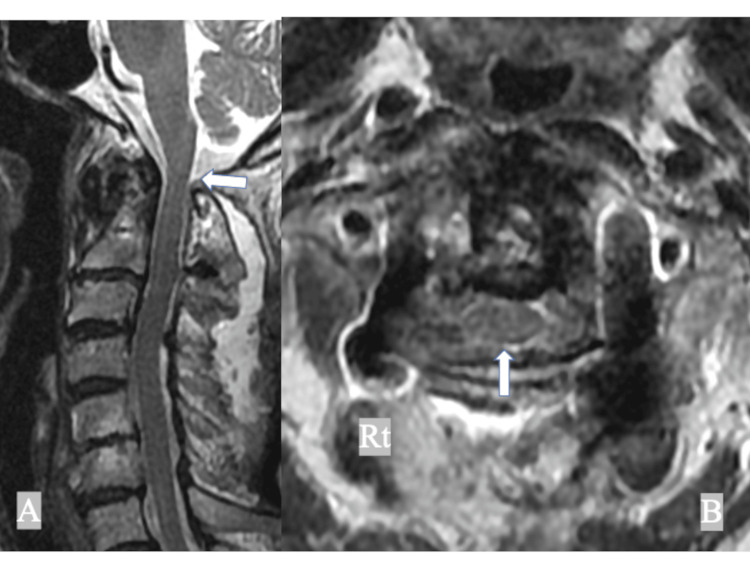
Postoperative MRI of the cervical spine showing (A, B) adequate decompression of the spinal cord at C1/2 (white arrows).

**Figure 8:**
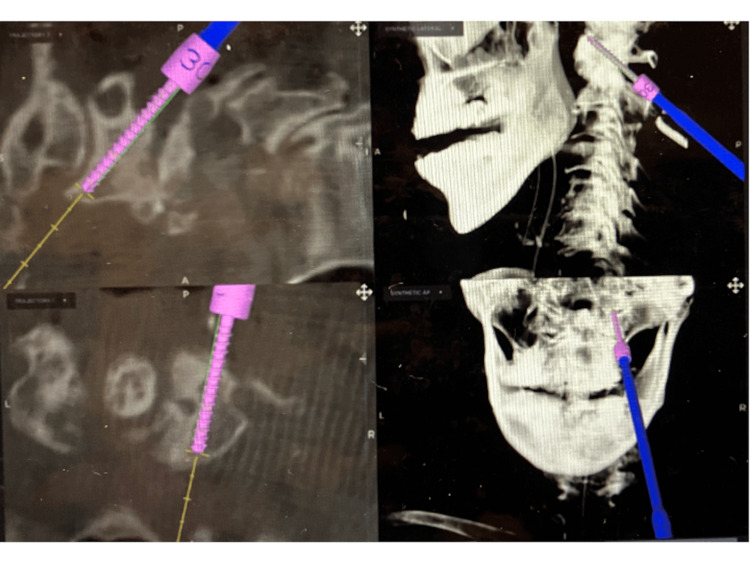
C1 lateral mass screw insertion under O-arm navigation guidance A 30 mm long screw (22 mm within the lateral mass) is inserted obliquely towards the cranioventral corner of the C1 lateral mass from the caudal side to the C2 nerve root on the right (ponticulus posticus side) under O-arm navigation guidance.

Postoperative radiographs and CT showed good reduction of AAS (Figures 5, 6) and MRI showed adequate decompression of the spinal cord at C1/2 (Figure 7). The right C1/2 lateral joint was exposed subperiosteally from the superior C2 lamina, and the caudal-dorsal surface of the C1 lateral mass was identified without direct manipulation of the C1-C2 venous plexus and C2 nerve root. The C1-C2 venous plexus and C2 nerve root were protected using the Penfield elevator and a hole for the C1 LMS was drilled with a 2-mm diamond burr, and a 30 mm long screw (22 mm length within the lateral mass) was inserted obliquely towards the cranioventral corner of the C1 lateral mass under O-arm navigation guidance (Figure 8) [15].

Postoperatively, occipitalgia, posterior neck pain, and numbness in both hands resolved, and the patient was discharged without complications. 

## Discussion

The "ponticulus posticus" means "little posterior bridge" in Latin and was described by Cleland et al. in 1861 [16]. Clinically, it refers to an abnormal bony bridge of the C1 posterior arch, and its pathology is generally considered to be ossification of the posterior atlantooccipital membrane (ligament), diagnosed incidentally regardless of age [1-3]. Its prevalence is about 16% in the population and it is not a rare variant entity, so it is sometimes combined with other upper cervical spine disorders.

AAS is a well-known unstable condition of the upper cervical spine resulting from various pathologies such as rheumatoid arthritis, trauma, os odontoideum, Down’s syndrome, and OA. Atlantoaxial fusion is a primary procedure for AAS, and among various methods, C1 LMS has recently become the most widely used technique since Goel-Harms’ reports. To date, six trajectories for C1 LMS insertion have been reported: (i) Goel and Laheri in 1994 [9], (ii) Harms and Melcher in 2001 [10], (iii) Tan et al. in 2003 (C1 PS via the posterior arch) [11], (iv) Lee et al. in 2006 (notch method) [14], (v) Wada et al. in 2016 (caudal to C2 nerve root approach) [15], and (vi) Lee et al. in 2021 (over-the-arch technique) [17].

The Goel-Harms direct lateral mass approach below the posterior arch sometimes troubles the surgeon with massive bleeding from the C1-C2 venous plexus and postoperative C2 nerve root irritation. To solve these problems, C1 LMS insertion via the posterior arch (C1 PS) has been proposed by Tan et al. [11], and supported by several authors due to its feasibility and strong fixation [14,16]. However, for safe insertion via the posterior arch, a thickness of the C1 posterior arch of >4 mm is required and approximately 19% of patients did not meet this criterion [18]. Wada et al. proposed an alternative C1 LMS insertion trajectory from the caudal side of the C2 nerve root in 2016, citing the advantages of this approach as follows: (i) subperiosteal exposure of the C2 lamina to the C1/2 lateral joint without direct manipulation of the C1-C2 venous plexus and C2 nerve root, and (ii) a long oblique screw trajectory in the C1 lateral mass from the caudal-dorsal to the cranial-ventral direction, which helped to avoid massive bleeding from the C1-C2 venous plexus and occipitalgia due to C2 nerve root irritation observed with the conventional Goel-Harms C1 LMS insertion and increasing fixation force [15]. Senoglu et al. [19] evaluated the anatomical and morphometric measurements of the C1 LMS trajectory inserted from the caudal side of the C2 nerve root proposed by Wada et al. [15], using the reconstructed 3D CT images of the atlas, and performed a detailed quantitative analysis of the optimal entry point, trajectory angle, screw length, and safety zone (the mean screw length within the lateral mass: 21± 2 mm) [19]. 

In the present case, we could not identify the PP on the right side at the first presentation due to advanced OA and AAS. 3D CTA could clearly show (i) the type of PP (complete or incomplete), (ii) the anatomical relationship between the VA and the posterior atlantal arch, and (iii) the thickness of the posterior arch below the PP. As the complete PP on the right side encircled the VA and the space between the VA and the thin posterior arch below the PP was very narrow in this case, the usual C1 LMS insertion through the posterior arch (C1 PS) or the notch trajectory had a significant risk of injuring the VA. Because the posterior height of the C1 lateral mass on the right side (PP side) was narrower than that on the left side due to the change in OA, we chose an alternative trajectory for the right C1 LMS insertion from the caudal side of the C2 nerve root (according to Wada et al. [15]) instead of the conventional Goel-Harms C1 LMS insertion, and finally we succeeded in avoiding the more invasive OCF. To the best of our knowledge, there are few reports on atlantoaxial posterior fusion in AAS with PP using the C1 LMS inserted from the caudal side of the C2 nerve root.

In addition, bilateral HRVA was detected by 3D CTA and we performed the C2 laminar screw and the extension of fixation with the C3 PSs. As HRVA is as common as PP and the prevalence of at least unilateral HRVA was reported to be 25.3% in an earlier systematic review [20], we should remember the possibility of the combined anatomical and vascular variations with PP and HRVA when planning C1-C2 posterior screw fixation in AAS. 

## Conclusions

To treat complex upper cervical disorders with AAS, PP, and HRVA, we should select the safest and most effective posterior screw constructs to avoid catastrophic complications and invasive OCF in an elderly patient. For C1-C2 posterior screw fixation in these patients, careful preoperative evaluation of the VA with 3D CTA is mandatory to avoid unexpected VA injury. Currently, we can choose several C1-C2 posterior screw trajectories depending on the surrounding vascular and osseous anatomy and select an optimal combination of screw constructs. C1 LMS insertion from the caudal side of the C2 nerve root may become a useful trajectory option among several approaches to the atlas in patients with AAS and PP.
